# Gene Structures, Classification, and Expression Models of the *DREB* Transcription Factor Subfamily in *Populus trichocarpa*

**DOI:** 10.1155/2013/954640

**Published:** 2013-11-13

**Authors:** Yunlin Chen, Jingli Yang, Zhanchao Wang, Haizhen Zhang, Xuliang Mao, Chenghao Li

**Affiliations:** State Key Laboratory of Forest Genetics and Tree Breeding, Northeast Forestry University, 26 Hexing Road, Harbin 150040, China

## Abstract

We identified 75 dehydration-responsive element-binding (*DREB*) protein genes in *Populus trichocarpa*. We analyzed gene structures, phylogenies, domain duplications, genome localizations, and expression profiles. The phylogenic construction suggests that the *PtrDREB* gene subfamily can be classified broadly into six subtypes (*DREB* A-1 to A-6) in *Populus*. The chromosomal localizations of the *PtrDREB* genes indicated 18 segmental duplication events involving 36 genes and six redundant *PtrDREB* genes were involved in tandem duplication events. There were fewer introns in the *PtrDREB* subfamily. The motif composition of *PtrDREB* was highly conserved in the same subtype. We investigated expression profiles of this gene subfamily from different tissues and/or developmental stages. Sixteen genes present in the digital expression analysis had high levels of transcript accumulation. The microarray results suggest that 18 genes were upregulated. We further examined the stress responsiveness of 15 genes by qRT-PCR. A digital northern analysis showed that the *PtrDREB17, 18,* and *32* genes were highly induced in leaves under cold stress, and the same expression trends were shown by qRT-PCR. Taken together, these observations may lay the foundation for future functional analyses to unravel the biological roles of *Populus' DREB* genes.

## 1. Introduction

Environmental stresses, such as drought, high salt, and low temperature, have adverse effects on plant growth and development. During evolution, plants established physiological and metabolic defense system responses to adverse conditions. In essence, stress induced the expression of specific genes and their products play a role in the plant's stress defense mechanism. Transcription factors (TFs) can upregulate a set of genes under their control by interacting with cis-elements present in the promoter region of targeted genes. The products act as regulatory proteins, consequently enhancing the stress tolerance of the plant. The dehydration-responsive element-binding (*DREBs*) protein TFs play an important role in regulating plant growth and the response to external environmental stresses.

In the plant kingdom, *DREB* is a large subfamily belonging to the APETALA2/ethylene-responsive element-binding protein (AP2/EREBP) family. *DREB* genes contain the highly conserved AP2/ERF DNA-binding domain [1]. The AP2/ERF domains, which consist of 50 to 60 amino acids, are found in proteins involved in a variety of regulatory mechanisms throughout the plant life cycle. The *DREB* subfamily play an important role in the resistance of plants to abiotic stresses by recognizing the dehydration-responsive element (DRE), which has a core motif of A/GCCGAC [2], and some members of this gene subfamily recognize the cis-acting element AGCCGCC, known as the GCC box [3]. The *DREB* subfamily TFs have been identified in various plant species, including *mangrove *[4], *soybean *[5], and *potato *[6]. The roles of *DREB *proteins in the plant's response to abiotic stress have also been extensively documented [7]. In the genomes of *Arabidopsis* [3], *Vitis vinifera *[8], and *rice *[9], 56, 36, and 57 AP2/ERF-related proteins, respectively, are encoded. Genetic and molecular approaches have been used in combination to characterize a series of *DREB *family regulatory genes involved in many different pathways, including genes related to cold, drought, high salinity, heavy metals, and abscisic acid (ABA) [4].

The characterization of the *DREB *subfamily of genes in *Populus* can aid the understanding of the molecular mechanisms of stress resistance and thus aid in the development of *Populus *varieties, using transgenic technology, with a greater tolerance to many adverse environments. Some *DREB *subfamily genes have been isolated from rice, *Arabidopsis,* and other plants [10], but they have not been isolated from *Populus*. Thus, their functions remain to be determined in *Populus*. The completion of the high-quality sequencing of the *Populus* genome [11] has provided an excellent opportunity for genome-wide analysis of genes belonging to specific gene families. Therefore, we present a comprehensive and specific analysis of gene structure, chromosome localization, and expression of the *Populus*' DREB subfamily for the first time. Here, we identified 75 *PtrDREB *genes in *Populus *using database searches and classified these genes according to their homology with known genes. We describe DREB subtypes more specifically and present novel information from different tissues and/or developmental stages. Some subtypes of this gene subfamily were differentially expressed under abiotic stress conditions. *PtrDREB *genes play an important role in the cross-talk of signaling pathways responding to different kinds of stress. We analyzed the phylogenetic relationships of the *DREB *genes in *Populus* and attempted the complete alignment of the subtypes. We examined gene structure and conserved motifs of *DREB *genes. Taken together, our results will be helpful in determining the functions of each *DREB *gene.

## 2. Materials and Methods

### 2.1. Database Search and Sequence Retrieval

The *P. trichocarpa* genome DNA database was downloaded from Phytozome (http://www.phytozome.net/). The database of the *A. thaliana DREB *subfamily was downloaded from the *Arabidopsis *Information Resource (TAIR, http://www.arabidopsis.org/, release 10.0). A local BLAST search was performed using the amino acid sequences of the AP2/ERF domains from *Arabidopsis *as the queries for the identification of the *DREB *genes from *Populus*. All of the located sequences were further manually analyzed to confirm the presence of an AP2 domain using the InterProScan program (http://www.ebi.ac.uk/Tools/InterProScan/). The *Arabidopsis At4g13040 *was used as an outlier.

### 2.2. Phylogenetic Analysis

A phylogenetic analysis was initially performed using all the *DREB *genes from *Arabidopsis *and *Populus*. To construct the phylogenetic trees, full-length *Arabidopsis *and *Populus *amino acid sequences were aligned using ClustalX 1.83 software [12] and manually edited using Jalview to reduce gaps [13]. The phylogenetic analysis was performed by the maximum parsimony method with 1,000 bootstrap replicates using MEGA 4 software [14].

### 2.3. Chromosome Localization

Each of the *DREB* genes' chromosomal position in *Populus* was identified and plotted using the Phytozome (http://www.phytozome.net/) and Joint Genome Institute (http://genome.jgi-psf.org/pages/blast.jsf?db=Poptr1_1) websites. This information is provided in Table 2. A schematic view of the chromosomes was reorganized by the most recent whole-genome duplication in Populus.

### 2.4. Exon/Intron Structure and Motif Analysis

The exon/intron organization for individual DREB genes was illustrated using the Gene structure display server (GSDS) program (http://gsds.cbi.pku.edu.cn/). The CDS and genome sequences of the *P. trichocarpa* genes were obtained from NCBI (http://www.ncbi.nlm.nih.gov/). The program MEME (v4.3.0) (http://meme.sdsc.edu/) was used to deduce 75 *Populus* DREB protein sequences.

### 2.5. EST Profiling and Microarray Analysis

The expression profile for each gene was obtained by a Digital Northern tool at PopGenIE (http://www.popgenie.org/) that generated a digital northern expression profile heat map based on the EST representations of 19 cDNA libraries derived from different tissues and/or developmental stages [15]. The heat map was visualized using the Heatmapper Plus tool at the Bio-Array Resource for Plant Functional Genomics (http://bar.utoronto.ca/ntools/cgi-bin/ntools_heatmapper_plus.cgi/) [16]. The microarray data for various tissues/organs available at NCBI Gene Expression Omnibus (GEO) database under the series accession number GSE6422 were used for the tissue-specific expression analysis.

### 2.6. Plant Treatment and qRT-PCR Analysis

For expression pattern analysis of the *Populus* DREB gene subfamily under abiotic stresses, plants were exposed to 42°C for 0, 0.5, and 1 h; 4°C for 0, 12, and 24 h; 200 mM NaCl for 0, 4, and 8 h; 100 *μ*M ABA for 0, 2, 4, and 6 h; and 4% PEG6000 for 0, 4, and 8 h. Young leaves were harvested at various time points. All samples were immediately frozen in liquid nitrogen and stored at −80°C until RNA isolation. 

Total RNA from leaf was extracted using the CTAB method. Synthesized cDNAs were used for qRT-PCR, which was performed using the TaKaRa ExTaq RT PCR Kit and SYBR green dye (TaKaRa, Dalian, China) in 96-well optical reaction plates (Applied Biosystems, USA). The results obtained for the different stages were standardized to the levels of the *actin* gene using the 2^−ΔΔCT^ method. We selected 15 *DREB* genes to observe tolerance under stress conditions in *Populus* via EST profiling and microarray analysis. We designed the primers for gene expression analysis using Primer Premier 5 to produce amplified lengths of 180 to 200 bp (Table 1).

## 3. Results and Discussion

### 3.1. Identification of *DREB* Subfamily TFs in *Populus*

**Figure 1:**
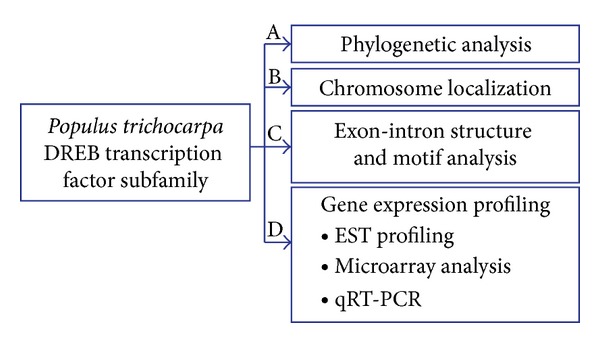
Integrated systems analysis workflow for elucidation of the role of *DREB *subfamily in the bioinformatics and data analysis in *Populus*. A: A phylogenetic analysis was performed using all the *DREB* subfamily amino acid sequences from *Arabidopsis* and *Populus* by MEGA 4 software. B: Each of the *DREB* genes' chromosomal position in *Populus* was using the Phytozome and Joint Genome Institute websites. C: The exon/intron organization for individual* DREB* genes was using the gene structure display server (GSDS) program and motif analysis was performed using the program MEME (v4.3.0). D: Gene expression profiling of *Populus DREB *subfamily was used to characterize differentially expressed genes.

To identify putative *DREB* genes in *Populus*, we performed a BLASTP search against *Populus *genome release v2.1 using *DREB *protein sequences from *Arabidopsis*. By removing the redundant sequences, 75 *DREB *genes were identified in the *Populus *genome. All *DREB *candidates were analyzed using the smart database (http://smart.embl-heidelberg.de/smart/set_mode.cgi?NORMAL=1) to verify the presence of AP2/ERF domains. Seventy-five *DREB *genes were used for the analysis of bioinformatics and gene expression profiling. The overall strategy used in this study was depicted in Figure 1 and presented in detail below. We designated *Populus DREB *genes as *PtrDREB *following the nomenclature proposed in a previous study [17]. *A*. *thaliana At4g13040 *was used as a query sequence and it includes an AP2/ERF-like domain sequence; however, its homology appears quite low in comparison with the other AP2/ERF genes. Therefore, this gene was also designated as an outlier. Detailed information on the *DREB *subfamily of genes in *Populus *and *Arabidopsis *are listed in Table 1.

### 3.2. Phylogenetic Relationships and Alignments of the *DREB* Subfamily in *Populus*

**Figure 2:**
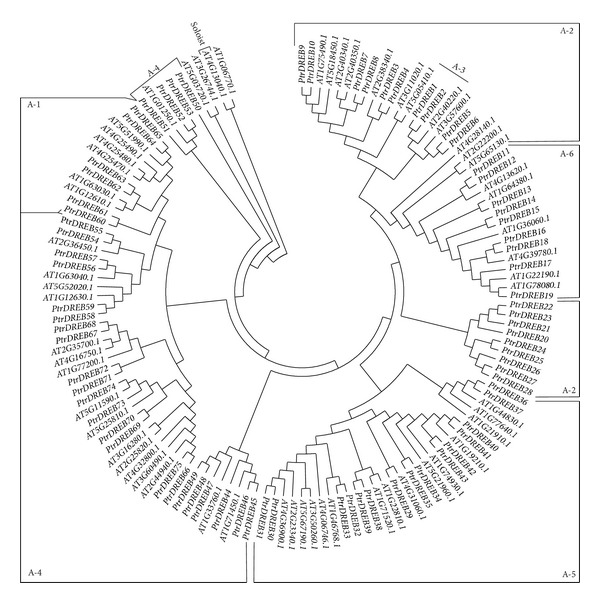
Relationships among *Populus DREBs* proteins after alignment with ClustalW. Proteins were allocated to six distinct subgroups of *DREB*, A-1 to A-6.

Based on the alignment of the AP2/ERF coding region protein sequences of all *Populus* and *Arabidopsis DREB *subfamily genes, 75 *DREB *subfamily genes of *Populus *were classified into six groups, A1, A2, A3, A4, A5, and A6, containing six, 17, two, 26, 15, and nine members, respectively. The phylogenetic trees of the *DREB *subfamilies of *Populus *and *Arabidopsis *are shown in Figure 2. The alignment analysis indicates that *DREBs* share high homology in the AP2/ERF domain (see Supplementary Figure 1; available online at http://dx.doi.org/10.1155/2013/954640) and contain a conserved WLG motif in the AP2/ERF domain of *Populus*. In the proteins encoded by *DREBs*, position 14 is normally valine and position 19 is glutamic acid. This region may play an important role in the recognition of different DNA-binding sites by the DRE and GCC box cis-elements of the *DREB *subfamily [3]. Also, the *DREBs *contain alanine at position 319 in the *β*-sheet. Group A-1 possesses a conserved C/SEV/LR amino acid sequence between V319 and E324, and this region is converted into an AEIR amino acid sequence in groups A-2, A-3, and A-6. In Group A-4, Ser-324 is crucial for the specific binding of the ERE element, and Ser-324/Ala-324 is crucial in Group A-5.

### 3.3. Chromosomal Locations of *DREB* Subgroups

**Figure 3:**
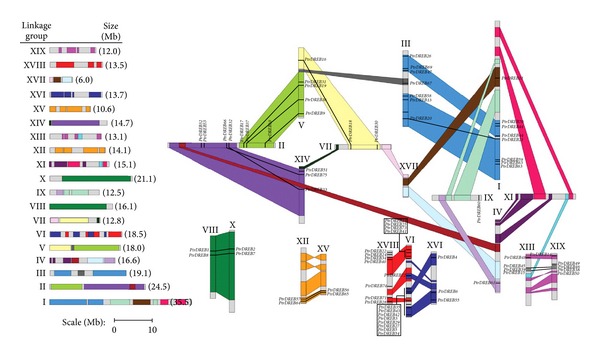
Locations of *P. trichocarpa DREB* genes on the chromosomes LGI-XIX. A schematic view of chromosome reorganization by recent whole-genome duplication in *Populus *is shown (adapted from [21]). Regions that are assumed to correspond to homologous genome blocks are shaded in the same color and connected with lines.

To examine the genomic distribution of *DREB* genes on *Populus *chromosomes, we identified their positions by a Phytozome database search. *In silico* mapping of the gene loci showed that 75 *Populus DREB *genes were mapped to linkage groups (LG) (Figure 3). Previous studies revealed that the *Populus *genome has undergone genome-wide duplications followed by multiple segmental duplications, tandem duplications, and transposition events [18]. It was very clear that three pairs of genes were arranged in tandem repeats, LG I (*PtrDREB63 *and *64*), LG II (*PtrDREB52 *and *53*), and LG VI (*PtrDREB43 *and *42*), and 18 pairs of genes were duplicated. The dN/dS ratios from the 18 segmental duplication pairs were less than 0.5. About 56% (42 of 75) of *Populus DREBs *were preferentially retained tandem duplications and multiple segmental duplication events. Tandem duplications and segmental duplications were relatively underrepresented in groups A-1 to A-6 with rates of 67% (4 of 6), 47% (8 of 17), 100% (2 of 2), 53% (14 of 26), 40% (5 of 15), and 89% (8 of 9), respectively. This finding corroborates previous findings that genes involved in transcriptional regulation and signal transduction are preferentially retained following duplications [19].

### 3.4. Gene Structure and Conserved Motifs of *Populus DREB* Genes

**Figure 4:**
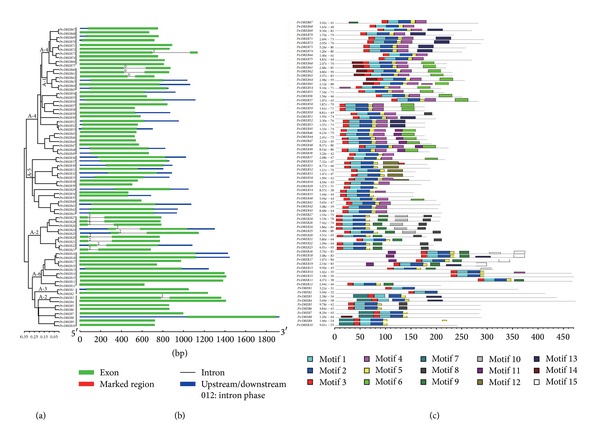
Phylogenomic analysis of 75 *DREB *genes in *P. trichocarpa *(a) with the integration of exon/intron structures (b) and MEME motifs (c). Exon/intron structure was obtained from the Gene Structure Display Server. Motifs were identified with the MEME software using the complete amino acid sequences of the *DREB* genes.

To gain further insights into the structural diversity of *Populus DREB *genes, we constructed a phylogenetic tree using the full-length *DREB *protein sequences of *Populus *(Figure 4(a)). We compared the exon/intron organization in the coding sequences of each *Populus DREB *gene (Figure 4(b)). All but nine *Populus DREB *members had no introns in their coding regions and the nine *Populus* DREB genes had one intron (*PtrDREB60*, *61*, *62*, *24*, *25*, *26*, *27*, *28, *and *3*). We predicted conserved motifs using MEME motif detection software that revealed the diversification of the *P. trichocarpa DREB *genes (Figure 4(c)), and 15 distinct motifs were identified (Table 3). The AP2/ERF domain consists of three **β**-sheet and one **α**-helix at the N termini [20, 21]. In this study, motif 3, specifying **β**-sheet strand 1; motif 1, specifying **β**-sheet strand 2 and 3; and motif 2, corresponding to the **α**-helix, were present in all of the *Populus' DREB *subfamily members. The CBF signature sequences (motif 14) were found in *DREB *Group A-1 [20, 21]. Alignment of the deduced amino acid sequences of *Arabidopsis DREB *Group A-1 TFs demonstrated significant similarity in the AP2/ERF binding domain and the CBF signature sequences [22]. These results suggest that the *P. trichocarpa DREB *Group A-1 share remarkable similarities at the amino acid sequence level with known CBF/DREB proteins of *Arabidopsis* and carry critical amino acids that are needed for binding to the CRT elements in the target genes. The CMIV domain (motif 7) was found in the *DREB *Group A-2. Alignment of the deduced amino acid sequences of *Arabidopsis DREB* Group A-2 TFs in the N-terminal region included the conserved motif CMIV-1 and the DNA-binding domain [23]. These results suggest that most of the closely related members in the phylogenetic tree shared a common motif composition with each other, suggesting functional similarities among the *DREB* proteins within the same subfamily.

### 3.5. EST Profiling and Microarray Analysis

**Figure 5:**
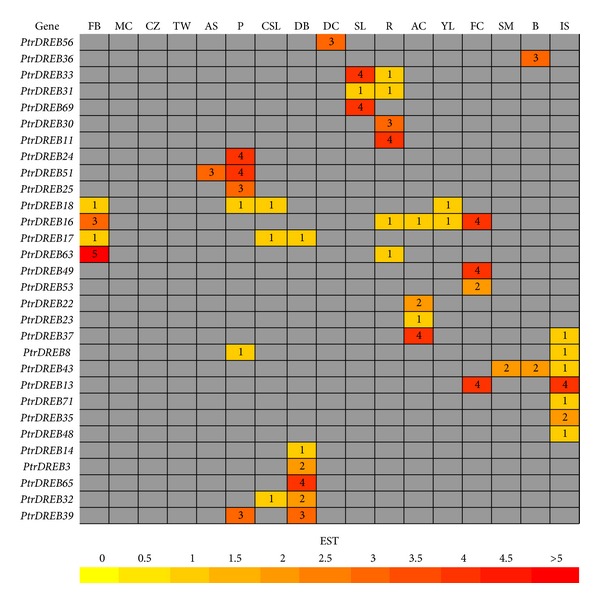
In silico EST analysis of* Populus DREB* genes. Color bar at bottom represents the frequencies of EST counts. FB: flower buds, MC: male catkins, CZ: cambial zone, TW: tension wood, AS: apical shoot, R: roots, CSL: cold stressed leaves, DB: dormant buds, DC: dormant cambium, SL: senescing leaves, P: petioles, AC: active cambium, YL: young leaves, FC: female catkins, SM: shoot meristem, B: bark, IS: imbibed seeds. Gene names are shown on the left.

The expression profile for each gene was obtained by the Digital Northern tool, which generates a digital northern expression profile heat map based on the ESTs, and is a useful additional means of inferring gene function by examining expression patterns based on the frequency of ESTs in libraries prepared from various tissues [15] (Figure 5). Such analysis yielded 32 *Populus DREB* genes in the available cDNA libraries. Of the 32 *DREBs *examined, 16 genes in the digital expression analysis had high transcript accumulation. A comparison of the digital northern expression analysis revealed that *PtrDREB16 *and* 63* had high transcript accumulation in flower buds, *PtrDREB51 *in apical shoots, *PtrDREB24*,* 25, *and* 51* in petioles, *PtrDREB39 *and *65* in dormant buds, *PtrDREB33 *and* 69* in senescing leaves, *PtrDREB11 *and* 30* in roots, *PtrDREB37* in active cambium, *PtrDREB71* in shoot meristem, *PtrDREB13*,* 16, *and* 49* in female catkins, *PtrDREB36 *in bark, and *PtrDREB13 *in imbibed seeds. On the whole, the remaining genes had low transcript accumulation in the different libraries examined. The low-abundance TFs had relatively low EST frequencies [24] and most of the *DREBs *were represented by only one single EST in the cDNA libraries. Nevertheless, this expression analysis demonstrated that most of the *DREBs *have a broad expression pattern across different tissues.

**Figure 6:**
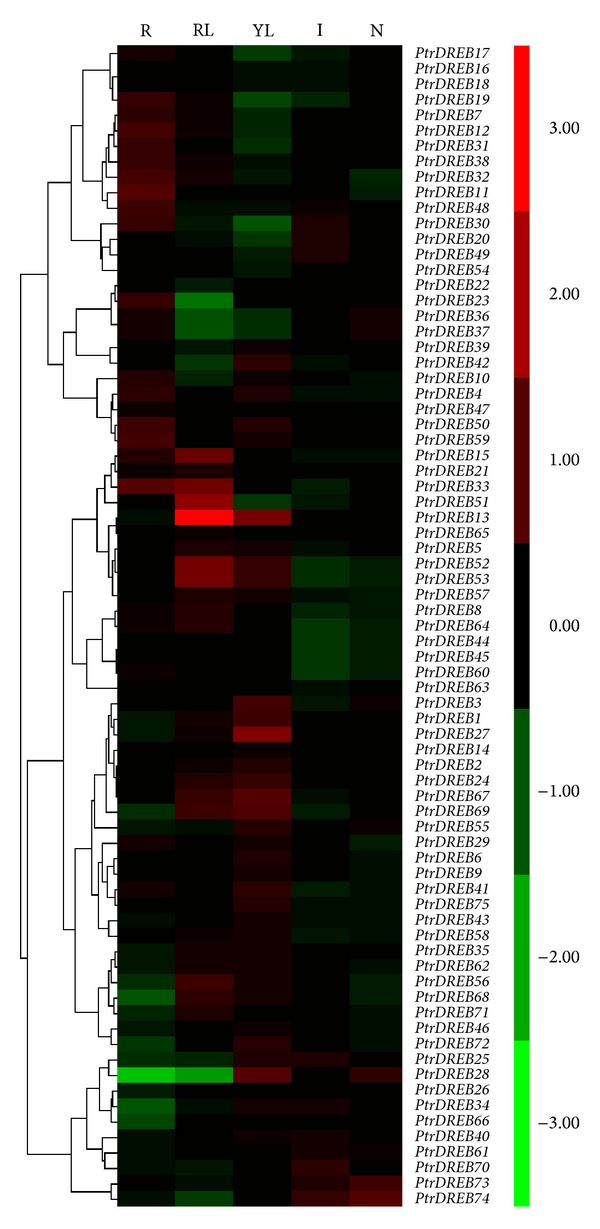
Expression profiles of *Populus DREB *genes across different tissues. Heat map showing hierarchical clustering of 75 *PtrDREB* genes across various tissues analyzed. The Affymetrix microarray data were obtained from NCBI Gene Expression Omnibus (GEO) database under the series accession number GSE16422. ML: mature leaf; YL: young leaf; R: root; I: internodes; N: nodes. Color scale represents log2 expression values, green represents low level, and red indicates high level of transcript abundances.

To gain more insights into the expression profiles of *DREB *genes, we then reanalyzed the previously published microarray data in *Populus*. We first investigated the global expression profiles of *DREB *genes by examining Affymetrix (GSE6422) [25] microarray data from Gene Expression Omnibus. Seventy-five *DREB *genes were included in GSE6422, and the *DREB *genes showed a distinct tissue-specific expression pattern (Figure 6). A comparison of the different tissues revealed that *PtrDREB13*,* 15*,* 33*,* 51, *and* 53* were overrepresented, however* PtrDREB23* and *28* were under-represented in mature leaves. *PtrDREB13*,* 27*,* 28*,* 67, *and* 69* were over-represented, however* PtrDREB30* was under-represented in young leaves. *PtrDREB20*,* 30*,* 49*,* 70, *and* 74* were over-represented in internodes. *PtrDREB28*,* 73, *and* 74* were over-represented in nodes. *PtrDREB7*,* 11, *and* 18* were over-represented, however* PtrDREB28* was under-represented in roots. On the whole, the remaining genes showed low-abundance transcription levels in the different tissues. EST profiling and microarray analysis showed that *PtrDREB28*,* 73*,* 74*,* 37, *and* 71* had high-abundance transcripts in the cambium. Previous studies using genome-wide transcriptional profiling in *Arabidopsis* revealed that stress-related and touch-inducible genes are upregulated in stem regions where secondary growth takes place [26].

### 3.6. Expression of *DREB* Genes under Abiotic Stress Conditions in *Populus*

**Figure 7:**
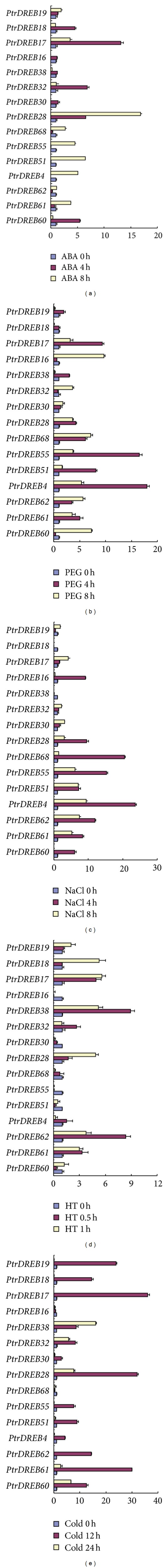
Expression analysis of fifteen selected *P. trichocarpa* DREB genes in mature leaves under ABA (a), drought stresses (b), salinity (c), high temperature (d), and low temperature (e) by qRT-PCR. The data were normalized using the *P. trichocarpa* actin gene. Standard deviations were derived from three replicates of each experiment.

Studies have previously been conducted to evaluate the expression of the *DREB Populus* genes in response to stresses such as low temperature, high temperature, salt, and dehydration [7]. However, the genes that were evaluated in our study were predicted to be candidate genes in *Populus *via EST profiling and microarray analysis. We have quantified the expression levels of the genes in leaf tissue after exposure to different abiotic stress conditions. The results provide an abundant set of information regarding the expression of these genes in *Populus* in response to low temperature, high temperature, ABA, salt, and dehydration (Figure 7). Among 15 selected *DREB* genes, there was evidence of induced expression under different abiotic stresses conditions, with the exception of *PtrDREB30*.

*PtrDREB60*, *61,* and *62*, which belong to the A-1 subgroup, were stress-inducible by low temperature, salt, and dehydration (Figure 7). In addition, *PtrDREB62* was stress-inducible by high temperature and *PtrDREB60* was stress-inducible by ABA. These findings are consistent with those of Dubouzet et al. (2003), who reported the increased expression of an A-1 subgroup gene (*OsDREB1*) of rice [10]. It was a major regulator of cold-stress responses in the *DREB1*/CBF (A-1) subgroup [7]. In a recent study, they indicated that ethylene signaling plays a negative role in the adaptation of *Arabidopsis* to freezing stress [26]. Additionally, some studies indicated that the *AmCBF2* was inducible by heavy metals (Pb^2+^ or Zn^2+^) [4].

*PtrDREB4* and *PtrDREB28* belong to the A-2 subgroup (Figure 7), and they were stress-inducible by ABA, salt, and dehydration. In addition, *PtrDREB28* was stress-inducible by high temperature and low temperature. This finding is consistent with those of Dubouzet et al. (2003) [10], who reported increased expression of an A-2 subgroup gene (*MsDREB2C*) of* Malus sieversii Roem *[27]. It was a major regulator of dehydration and heat shock responses in the DREB2 subgroup [7]. This indicated that ethylene signaling plays a negative role in the adaptation of *Arabidopsis* to freezing stress [26]. The oxidative stress tolerance of DREB2C-overexpressing transgenic *Arabidopsis* plants was regulated by heat shock factor A3 (*HsfA3*) and *HsfA3* is regulated at the transcriptional level by *DREB2* [28]. 

*PtrDREB51*, *55,* and *68* belong to the A-4 subgroup, and they were stress-inducible by salt and dehydration. However, *PtrDREB51*, *55,* and *68 *were down represented by high temperature *PtrDREB68* was down represented by low temperature. Genes *PtrDREB30*, *32,* and *38* belonged to subgroup A-5. *PtrDREB32* was stress-inducible by cold and ABA, whereas *PtrDREB38* was induced by hot and cold temperatures. *PtrDREB30* was down represented by high temperature, whereas *PtrDREB38* was down represented by salt. *PtrDREB16*, *17*, *18, *and *19* belonged to the A-6 subgroup. *PtrDREB16 *was induced by drought and high salt, and *PtrDREB17 *was induced by drought, high temperature, low temperature, and ABA treatment. *PtrDREB18 *was induced by high temperature, low temperature, and ABA. In addition, *PtrDREB19 *was only induced by low temperature. *PtrDREB18* was down represented by by salt, whereas *PtrDREB16* was down represented by high temperature and cold temperature (Figure 7). In our study, a digital northern analysis showed that the genes *PtrDREB17*, *18, *and *32 *were expressed in cold-stressed leaves, and showed the same expression trends based on qRT-PCR. The expression patterns of* Populus DREB *genes detected by qRT-PCR are generally consistent with microarray analyses and digital northern analyses.

Some studies indicate that the *HARDY *(*At2g36450*) gene belongs to the A-4 subgroup. The overexpression of the *HRD* gene for the improvement of water-use efficiency is coincident with drought resistance in rice. The analogous genes *At2g36450 *and *PtrDREB68* were stress-inducible in drought [29]. The homologous genes, RAP2.4 (*At1g78080*) and *PtrDREB19* and RAP2.*4B *(*AT1G22190*) and *PtrDREB17*, were expressed in response to dehydration, high salinity, and heat [30, 31]. Overexpression or mutation of *RAP2*.4 and *RAP2.*4B in *Arabidopsis *acts at or downstream of a point of convergence for light and ethylene signaling pathways that coordinately regulates multiple developmental processes and stress responses [30]. It is noteworthy that the expression of several other genes associated with lipid transport was altered in the *RAP2*.4 and *RAP2*.4B overexpression lines, further supporting the link between *DREB *TFs and adaptive alterations in lipid metabolism [31]. Hence, we think that the *PtrDREB16 *and *17 *TFs are probably associated with enhanced drought tolerance by modulating the wax biosynthetic pathway.

## 4. Conclusions

Understanding the plant *DREB* subfamily is important for elucidating the mechanisms of a variety of stress responses. Therefore, we present a comprehensive and specific analysis of gene structure, chromosome localization, and expression of the *Populus DREB *subfamily for the first time. We predicted *P. trichocarpa DREB *gene expression and function through comparisons with similar genes that have been well studied in model or other plants. The chromosomal localizations of the *PtrDREB *genes involved in transcription regulation and signal transduction are preferentially retained following duplications. The conserved motif composition of *PtrDREB *genes were highly conserved in the same subtype. EST profiling and microarray analysis of this gene subfamily from different tissues and/or developmental stages showed the same expression trends based on qRT-PCR. The results in the present study indicate that *DREBs* function as important transcriptional activators and may be useful in improving plant tolerance to abiotic stresses. Taken together, these observations may lay the foundation for future functional analyses of *Populus DREB *genes to unravel their biological roles.

## Supplementary Material

Alignment of the predicted amino acid sequence from selected members of the DREB family in Populus trichocarpa. Amino acids are expressed in the standard single letter code. the PtrDREB gene subfamily can be classified broadly into six subtypes (DREB A-1 to A-6) in Populus. The AP2/ERF domain consists of three *β*-sheet and one *α*-helix at the N termini. Arrows designate the highly conserved 14th valine (V14) and 19th glutamic acid (E19).

## Figures and Tables

**Table 1:** The primers of *DREB* genes were generated in Primer 5 for qRT-PCR.

Name	Sequence (5′-3′)
PtrDREB4F	GTATTGAGGGGAGAAATGGATGG
PtrDREB4R	CATATCATGGTCGGAAGACAAGC
PtrDREB16F	AATCTTGCTACCACCACATCACAGT
PtrDREB16R	ATGCCTCCGCCTGACTCCTCTAT
PtrDREB17F	ACTCTGGCTTGGCACATTTGAC
PtrDREB17R	GGCTTGTATTCGCCGATGTAGGA
PtrDREB18F	GGCTCCAAAACCTGTCCCTATGA
PtrDREB18R	CCCAATGTCTCTGCCTCACTCCT
PtrDREB19F	GAGGAGGCGGCTTTGGCTTAT
PtrDREB19R	AACCGAGGAATGGAGAGGCTTG
PtrDREB28F	CAGTCAAAAAAGTTCAGAGGGGT
PtrDREB28R	CTCTTCTGCTGTTTCAAATGTGC
PtrDREB30F	GCATGTAACGGTAGAAAGGAGGGGG
PtrDREB30R	AGATTGGCGGTAGATCAAGAGTG
PtrDREB32F	AGAAGGAAGTCATCAACAAGGGG
PtrDREB32R	ATTTGGTGCAGGCTGAGGCAA
PtrDREB38F	GTGAGAGGCAATACAAGGGGA
PtrDREB38R	CGCTACTGGTGTTGAGTAGGAA
PtrDREB51F	TGACCCGACCTCAAACTCTCCAG
PtrDREB51R	TCAGACACCCATTTCCCCCACCT
PtrDREB55F	GATTCTCAACCAACCAAAACCTC
PtrDREB55R	GGCTCTCTAATTTCAGACACCCA
PtrDREB60F	GAAGAAGAACAAAGCGGGAAGGA
PtrDREB60R	CATTTCTGGGCTCTTGAAGGTCC
PtrDREB61F	GCAGGAAGGAAGAAGTTCAAGGA
PtrDREB61R	GGCTAGTGAAGGTCCCTAACCAAAT
PtrDREB62F	TCTTCTTTCTCCGATAGCAGCAC
PtrDREB62R	CACCCTATTGTTACCATTCCTCT
PtrDREB68F	TCTAAGCGAAACCAAGACCCGAA
PtrDREB68R	TTTGCCCCATTGACGCATTCT
ActinF	CATCAAAGCATCGGTGAGGTC
ActinR	GTTGCCATCCAGGCTGTCC

**Table 2:** The *DREB* genes identified from the *P.  trichocarpa* genome.

Gene symbol	Gene Locus	PF00847 AP2 domain	*Arabidopsis *ortholog locus
PtrDREB1	POPTR_0008s07120	102–151	AT1G12610.1
PtrDREB2	POPTR_0010s19370	147–196	AT1G63030.1
PtrDREB3	POPTR_0006s10510	67–125	AT4G25470.1
PtrDREB4	POPTR_0016s13380	74–132	AT4G25480.1
PtrDREB5	POPTR_0006s05320	28–78	AT4G25490.1
PtrDREB6	POPTR_0016s05360	29–78	AT5G51990.1
PtrDREB7	POPTR_0010s19100	78–128	AT1G75490.1
PtrDREB8	POPTR_0008s07360	78–128	AT2G38340.1
PtrDREB9	POPTR_0005s25470	43–92	AT2G40340.1
PtrDREB10	POPTR_0002s03090	40–89	AT2G40350.1
PtrDREB11	POPTR_0001s32250	285–333	AT3G11020.1
PtrDREB12	POPTR_0017s08250	276–324	AT3G57600.1
PtrDREB13	POPTR_0003s13910	107–155	AT5G05410.1
PtrDREB14	POPTR_0019s13330	232–281	AT5G18450.1
PtrDREB15	POPTR_0013s13920	232–281	AT2G40220.1
PtrDREB16	POPTR_0005s07900	173–222	AT1G01250.1
PtrDREB17	POPTR_0002s09480	163–211	AT1G12630.1
PtrDREB18	POPTR_0007s05690	173–222	AT1G33760.1
PtrDREB19	POPTR_0005s16690	120–168	AT1G63040.1
PtrDREB20	POPTR_0003s17830	7–56	AT1G71450.1
PtrDREB21	POPTR_0001s14720	46–95	AT1G77200.1
PtrDREB22	POPTR_0018s02280	7–56	AT2G25820.1
PtrDREB23	POPTR_0006s27710	7–56	AT2G35700.1
PtrDREB24	POPTR_0018s01680	5–55	AT2G36450.1
PtrDREB25	POPTR_0006s26990	21–71	AT2G44940.1
PtrDREB26	POPTR_0003s02750	5–55	AT3G16280.1
PtrDREB27	POPTR_0006s06870	5–55	AT3G60490.1
PtrDREB28	POPTR_0018s13130	5–55	AT4G16750.1
PtrDREB29	POPTR_0006s08000	22–71	AT4G32800.1
PtrDREB30	POPTR_0007s10750	22–71	AT5G11590.1
PtrDREB31	POPTR_0005s18430	22–71	AT5G25810.1
PtrDREB32	POPTR_0002s12550	35–84	AT5G52020.1
PtrDREB33	POPTR_0014s02530	35–84	AT1G19210.1
PtrDREB34	POPTR_0018s00700	14–63	AT1G21910.1
PtrDREB35	POPTR_0006s14110	14–63	AT1G22810.1
PtrDREB36	POPTR_0005s15830	31–80	AT1G44830.1
PtrDREB37	POPTR_0002s08610	26–76	AT1G46768.1
PtrDREB38	POPTR_0019s10220	29–78	AT1G71520.1
PtrDREB39	POPTR_0013s10420	20–70	AT1G74930.1
PtrDREB40	POPTR_0018s08320	18–68	AT1G77640.1
PtrDREB41	POPTR_0006s23480	23–73	AT2G23340.1
PtrDREB42	POPTR_0006s14090	16–66	AT3G50260.1
PtrDREB43	POPTR_0006s14100	16–66	AT4G06746.1
PtrDREB44	POPTR_0001s18180	15–65	AT4G31060.1
PtrDREB45	POPTR_0013s10340	14–64	AT4G36900.1
PtrDREB46	POPTR_0019s10420	11–62	AT5G21960.1
PtrDREB47	POPTR_0003S05300	16–66	AT5G67190.1
PtrDREB48	POPTR_0013s10330	49–99	AT1G36060.1
PtrDREB49	POPTR_0019s10430	15–65	AT1G64380.1
PtrDREB50	POPTR_0019s09530	36–84	AT1G78080.1
PtrDREB51	POPTR_0014s09540	38–88	AT2G22200.1
PtrDREB52	POPTR_0855s00200	20–68	AT4G13620.1
PtrDREB53	POPTR_0002s17330	20–68	AT4G28140.1
PtrDREB54	POPTR_0006s02180	44–93	AT4G39780.1
PtrDREB55	POPTR_0016s02010	50–98	AT5G65130.1
PtrDREB56	POPTR_0015s13840	84–132	AT1G22190.1
PtrDREB57	POPTR_0012s13880	115–163	AT4G13040.1
PtrDREB58	POPTR_0003s12120	14–61	
PtrDREB59	POPTR_0001s08740	14–61	
PtrDREB60	POPTR_0009s14990	57–108	
PtrDREB61	POPTR_0004s19820	59–110	
PtrDREB62	POPTR_0001s08710	66–116	
PtrDREB63	POPTR_0001s08720	62–111	
PtrDREB64	POPTR_0012s13870	85–135	
PtrDREB65	POPTR_0015s13830	79–129	
PtrDREB66	POPTR_0002s14210	94–143	
PtrDREB67	POPTR_0003s07700	96–146	
PtrDREB68	POPTR_0001s15550	71–122	
PtrDREB69	POPTR_0003s04920	87–137	
PtrDREB70	POPTR_0001s18800	54–103	
PtrDREB71	POPTR_0018s09270	69–119	
PtrDREB72	POPTR_0006s17670	67–117	
PtrDREB73	POPTR_0006s25500	59–109	
PtrDREB74	POPTR_0018s00270	59–109	
PtrDREB75	POPTR_0014s05500	90–139	

**Table 3:** Motif sequences of *DREB* genes identified in *P. trichocarpa* by MEME tools.

Motif	Width	Best possible match
1	21	WGKWVCEIREPRKKSRIWLGT
2	24	FPTPEMAARAHDVAALCIKGDSAI
3	11	KHPVYRGVRMR
4	21	LPVPASTSPRDIQAAAASAAA
5	8	LNFPDLVH
6	25	EEALFDMPNLLVDMAGGMLLSPPRI
7	29	GDGGNKPVRKVPAKGSKKGCMKGKGGPEN
8	15	EDHHIEQMIEELLDR
9	18	YKPLHSSVDAKLQAICQS
10	25	HIGVWQKKAGSRSSSNWVMKVELGN
11	15	GPITVRLSPSQIQAI
12	30	DMSAASIRKRATEVGAHVDAIETALNHHHH
13	29	STSSLTSLVSLMDLSSQEEELCEIVELPS
14	21	EVMLASRNPKKRAGRKKFRET
15	21	FESGNFMLQKYPSYEIDWASI
