# Supplementary material for: Gene Structures, Classification, and Expression Models of the DREB Transcription Factor Subfamily in Populus trichocarpa
Source: ScientificWorldJournal. 2013 Nov 13;2013:954640. doi: 10.1155/2013/954640 (PMC3845248; doi:10.1155/2013/954640)
Supplement: Supplementary file 1 — Alignment of the predicted amino acid sequence from selected members of the DREB family in Populus trichocarpa. Amino acids are expressed in the standard single letter code. the PtrDREB gene subfamily can be classified broadly into six subtypes (DREB A-1 to A-6) in Populus. The AP2/ERF domain consists of three β-sheet and one α-helix at the N termini. Arrows designate the highly conserved 14th valine (V14) and 19th glutamic acid (E19). [file 954640.f1.pdf]

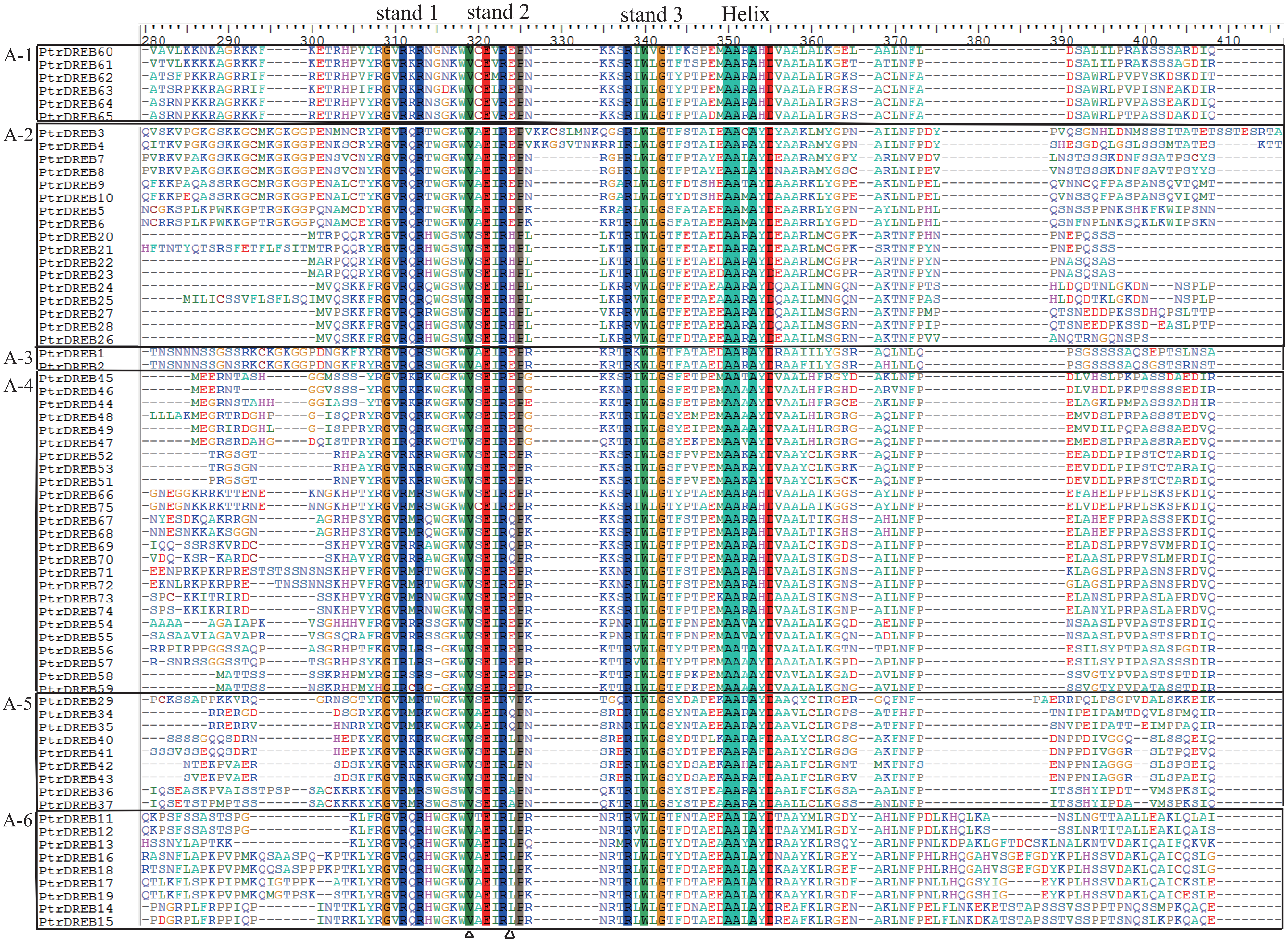
stand 1
stand 2
stand 3
Helix
A-1
A-2
A-3
A-4
A-5
A-6
PtrDREB60
PtrDREB61
PtrDREB62
PtrDREB63
PtrDREB64
PtrDREB65
PtrDREB3
PtrDREB4
PtrDREB7
PtrDREB8
PtrDREB9
PtrDREB10
PtrDREB5
PtrDREB6
PtrDREB20
PtrDREB21
PtrDREB22
PtrDREB23
PtrDREB24
PtrDREB25
PtrDREB27
PtrDREB28
PtrDREB26
PtrDREB1
PtrDREB2
PtrDREB45
PtrDREB46
PtrDREB44
PtrDREB48
PtrDREB49
PtrDREB47
PtrDREB52
PtrDREB53
PtrDREB51
PtrDREB66
PtrDREB75
PtrDREB67
PtrDREB68
PtrDREB69
PtrDREB70
PtrDREB71
PtrDREB72
PtrDREB73
PtrDREB74
PtrDREB54
PtrDREB55
PtrDREB56
PtrDREB57
PtrDREB58
PtrDREB59
PtrDREB29
PtrDREB34
PtrDREB35
PtrDREB40
PtrDREB41
PtrDREB42
PtrDREB43
PtrDREB36
PtrDREB37
PtrDREB11
PtrDREB12
PtrDREB13
PtrDREB16
PtrDREB18
PtrDREB17
PtrDREB19
PtrDREB14
PtrDREB15
